# Beyond “Big Eaters”: The Versatile Role of Alveolar Macrophages in Health and Disease

**DOI:** 10.3390/ijms22073308

**Published:** 2021-03-24

**Authors:** Miriam Hetzel, Mania Ackermann, Nico Lachmann

**Affiliations:** 1 Institute of Experimental Hematology, Hannover Medical School, 30625 Hannover, Germany; hetzel.miriam@mh-hannover.de (M.H.); Ackermann.Mania@mh-hannover.de (M.A.); 2 REBIRTH Research Center for Translational and Regenerative Medicine, Hannover Medical School, 30625 Hannover, Germany; 3 Department of Pediatric Pneumology, Allergology and Neonatology, Hannover Medical School, 30625 Hannover, Germany; 4 Biomedical Research in Endstage and Obstructive Lung Disease Hannover (BREATH), Member of the German Center for Lung Research (DZL), 30625 Hannover, Germany; 5 Cluster of Excellence RESIST (EXC 2155), Hannover Medical School, 30625 Hannover, Germany

**Keywords:** alveolar macrophage, surfactant, pulmonary infections, asthma, fibrosis, tolerogenic potential, pulmonary alveolar proteinosis, monocytes

## Abstract

Macrophages act as immune scavengers and are important cell types in the homeostasis of various tissues. Given the multiple roles of macrophages, these cells can also be found as tissue resident macrophages tightly integrated into a variety of tissues in which they fulfill crucial and organ-specific functions. The lung harbors at least two macrophage populations: interstitial and alveolar macrophages, which occupy different niches and functions. In this review, we provide the latest insights into the multiple roles of alveolar macrophages while unraveling the distinct factors which can influence the ontogeny and function of these cells. Furthermore, we will highlight pulmonary diseases, which are associated with dysfunctional macrophages, concentrating on congenital diseases as well as pulmonary infections and impairment of immunological pathways. Moreover, we will provide an overview about different treatment approaches targeting macrophage dysfunction. Improved knowledge of the role of macrophages in the onset of pulmonary diseases may provide the basis for new pharmacological and/or cell-based immunotherapies and will extend our understanding to other macrophage-related disorders.

## 1. Introduction

**Figure 1:**
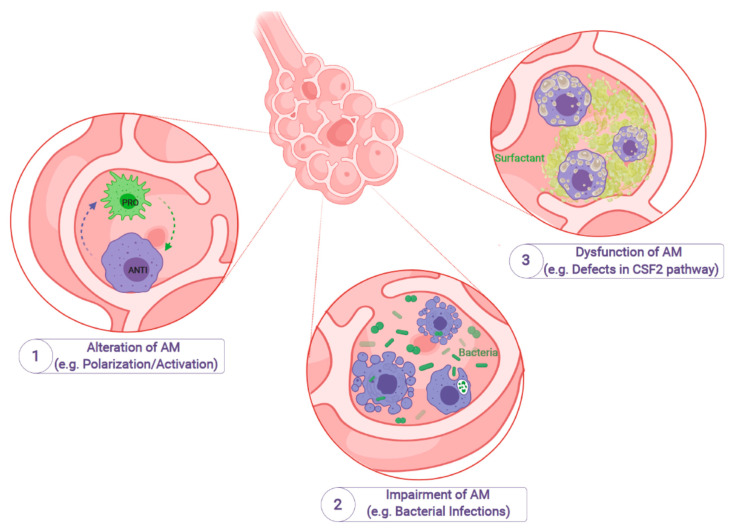
Impairment of alveolar macrophages leads to specific diseases or infections. Alveolar macrophages (AM) are the most abundant cell type in the bronchioalveolar space. Given the high plasticity of AM and the possibility to switch between different activation stages, alteration of AM can lead to a nonfunctional polarization/activation and subsequent alterations in, e.g., maintaining tissue homeostasis (1). Furthermore, genetic and environmental factors can also influence the antimicrobial function of AM, which can lead to life-threatening pulmonary infections (e.g., bacterial airway infections) (2). Dysfunction of AM due to genetic mutations or other factors can also alter signaling pathways (e.g., the CSF2 pathway), which ultimately can lead to impaired tissue homeostasis, e.g., clearance of surfactant material, as seen in Pulmonary Alveolar Proteinosis (3) (Created with BioRender.com).

Tissue resident macrophages (TRM) have recently been highlighted as key players in the tissue homeostasis of various organs. Within the lung, several subsets of macrophages exist, such as interstitial macrophages (IM) or alveolar macrophages (AM). In fact, nonalveolar IM are located primarily in the lung tissue around blood vessels or peribronchial and significantly contribute to lung immune homeostasis and other tolerogenic cellular pathways. In contrast, the classical AM reside in the bronchioalveolar space and represent an important cell type in lung surfactant homeostasis, pulmonary host defense, and tissue tolerance (Figure 1). In the alveolar space, AM are the best characterized and the most abundant cell type and due to their crucial role direct impairment in both, function and ontogeny of AM was noted to be important in the onset and progression of a variety of lung diseases [1]. Of note, while IM also fulfil manifold important functions in lung homeostasis (as reviewed elsewhere [2]), we here focus on the contribution of AM to the onset and progression of certain lung diseases.

Under healthy, steady-state conditions, AM are predominantly derived from Myb-independent yolk sac progenitors, which develop before the emergence of hematopoietic stem cells (HSC). Studies in the murine system showed that erythro-myeloid progenitors (EMP) give rise to premacrophages, which colonize the individual tissues, including the lung, before birth and give rise to TRM populations [3]. In the human system, recent insights suggest a similar developmental trajectory, which follows a spatiotemporal dynamic of TRMs [4]. In the absence of inflammation and tissue damage, alveolar macrophages are long lived and can self-maintain their cell pool by local proliferation, with only minor contribution of adult monocytes over time. However, inflammatory processes can trigger the recruitment of monocytes to the lung, where they potentially participate in pathogenic processes. Under which circumstances these monocytes reside in the lungs and to which degree they finally resemble resident AMs is still under debate. In this context, the “niche model” suggests that strong triggers from the lung microenvironment imprint the AM phenotype on the infiltrating monocytes over time. Indeed, recent work by Evren and colleagues demonstrated that human monocytes are able to adopt an AM phenotype over time and reside long-term in the lung of immunodeficient mice [5].

**Figure 2:**
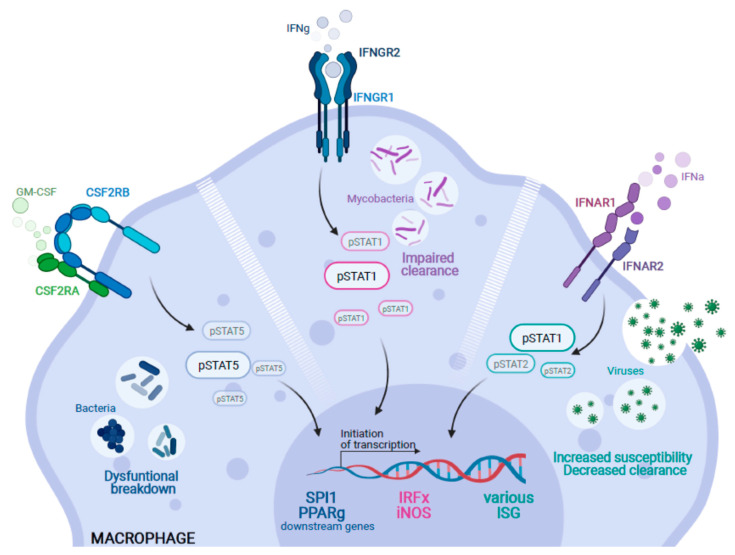
Genetic alterations causing impairment of alveolar macrophages in the context of pulmonary infection. Inborn errors in various genes can lead to alveolar macrophage dysfunction. (I) Following binding of GM-CSF, the CSF2R complex (CSF2RA/B) initiates downstream signaling by phosphorylation of STAT5 (pSTAT5) to activate important target genes such as *SPI1* or *PPARγ*. Thus, mutations in either *CSF2RA* or *CSF2RB* lead to a block of GM-CSF signaling which can impair proper breakdown of bacteria. (II) Similarly, IFNγ activates the IFNgR1-IFNgR2 signaling complex and induces downstream phosphorylation of STAT1 (pSTAT1) and the subsequent activation of target genes such as members of the Interferon-regulatory factors (IRF) or iNOS. An impairment of this signaling cascade leads to the impaired clearance of mycobacterial infections and the clinical symptoms of Mendelian Susceptibility to Mycobacterial Disease. (III) Moreover, loss of function mutations in type-I interferon receptors (IFNAR1/R2) can impair the phosphorylation of STAT1 and 2 (pSTAT1/2) and their respective downstream signaling. This results in an increased susceptibility and decreased clearance capacity of macrophages against viral pathogens (Created with BioRender.com).

Genetic defects, environmental factors, drugs and other determinants can influence both the ontogeny and function of AM, leading to a variety of diseases. In fact, recent research investigating the driving factors of patient’s susceptibility to pulmonary infections proved that signaling pathways like GM-CSF, or type I and/or II interferon signaling can be responsible for impaired development and/or function of AM [6,7] (Figure 2). Furthermore, other factors like dust and smoke or paralyzing agents can have a negative effect on the functionality of AMs. In our review, we will provide an overview of the different functions of AM as well as recent insights into various factors driving the onset and progression of lung diseases by either absent or dysfunctional AM. Moreover, we will discuss recent developments in the therapeutic targeting of AM where a special attention will be dedicated to the application of stem cell- or macrophage-based cell therapeutic strategies, highlighting the promising potential of utilizing the field of cell-based immunotherapies to combat AM-driven lung diseases.

## 2. The Implication of AMs in Lung Diseases

As mentioned before, AM fulfil several important functions in the lung niche that can be roughly divided into three main categories: surfactant homeostasis, the prevention of infections and the maintenance of tissue homeostasis. However, several studies suggest that AM are bad at “multitasking”, and if engaged in a certain activity they cannot sufficiently fulfill their other functions. For example, macrophages show a reduced antimicrobial function upon efferocytosis of apoptotic cells [8]. Similarly, exposure to fine particulate matter decreases AM cytokine secretion and phagocytosis of pathogens, which impairs pulmonary immunity [9,10,11,12]. Given the importance of all these functions for tissue functionality, the absence, malfunction or hyperactivation of AM can result in several diseases.

### 2.1. Missing or Malfunctional AM Are Key Drivers for Pulmonary Alveolar Proteinosis

The importance of AM for the lung surfactant homeostasis is most evident when functional AM are absent, which results in a disease referred to as pulmonary alveolar proteinosis (PAP). PAP is a heterogenous group of diseases, which are characterized by severe accumulation of surfactant in the lung and consequently severe respiratory failure [13]. Except for congenital PAP, which is caused by abnormal surfactant production, the pathophysiology of PAP is associated with the absence and malfunction of AM. Given their unique features, AM are essential in degrading surfactant, which is constantly produced by alveolar epithelial type II cells in order to facilitate lung function and gas exchange. The accumulation of the surfactant blocks the influx of air to the alveoli and prevents oxygen from passing through into the blood, resulting in dyspnea.

PAP can be categorized into: (I) primary PAP, which is caused by defects in the GM-CSF signaling axis that affects different stages of AM development as well as function and can be further divided into (I.I) autoimmune and (I.II) hereditary PAP; and (II) secondary PAP, which is mainly caused by reduced AM numbers due to other diseases. Moreover, there are also some clinical cases of PAP in which the underlying mechanism remains unknown, which are then categorized as (III) unclassified PAP. Although there are mechanistically distinct causes for the distinct forms of PAP, all of them are related to AM as the central cell type in lung homeostasis [13].

Autoimmune PAP is caused by autoantibodies against GM-CSF, the most crucial signal for AM maturation and function [14]. However, not all individuals with detectable GM-CSF autoantibodies develop PAP, as these antibodies have to target multiple epitopes throughout the GM-CSF protein, possess a high binding-capacity and effectively neutralize GM-CSF in higher concentrations to block the GM-CSF signaling axis and lead to the development of PAP [13]. Such autoantibodies can be found in autoimmune PAP patients and the infusion of patient-derived autoantibodies recapitulated the disease phenotype in nonhuman primates [15]. Similarly, the complete knockout of GM-CSF in *Csf2^−/−^* mice causes PAP-like symptoms, a discovery that led to the elucidation of the pathophysiology of PAP [16].

In hereditary PAP, the next player in the GM-CSF signaling axis is affected, which is the GM-CSF receptor (CSF2R). The CSF2R is a high affinity heterodimeric receptor complex composed of a ligand-binding α chain (CD116, encoded by *CSF2RA*) and a signal-transducing β chain (CD131, encoded by *CSF2RB*) that is shared with the receptors for IL-3 and IL-5 and therefore referred to as common β chain [17]. Mutations in both *CSF2RA* [18] and *CSF2RB* [19,20] have been identified in PAP patients and knockout mouse models for both genes exist, which recapitulate the disease phenotype observed in patients [21,22]. However, additional factors seem to influence disease development and progression, as relatives from one family with identic mutations can vary considerably in disease severity and clinical presentation [23]. While it is clear that AM have a pivotal role for the pathogenesis of PAP, the exact mechanism is versatile. It is clear that GM-CSF is a crucial player for the development of AM [14]. However, studies in *Csf2^−/−^* and *Csf2rb^−/−^* mice have shown that absent GM-CSF signaling does not lead to complete absence of AM [16,21]. These mice harbor malfunctioning AM, called foam cells, that take up massive amounts of surfactant, but in turn are not able to degrade and, thus, accumulate surfactant lipid and protein-laden vesicles in the cytoplasm. Recent studies suggest that the disease-causing component of the surfactant is cholesterol rather than phospholipids and surfactant proteins [24]. The surfactant is usually composed of 80% polar phospholipids, 10% neutral lipids (mainly cholesterol) and 10% surfactant proteins. However, in both PAP patients and in murine PAP models, the amount of cholesterol in relation to phospholipids in the surfactant is increased. One possible explanation could be cholesterol degradation defects in AM, which accumulate intracellular cholesterol and thus are unable to take up additional cholesterol, which then accumulates in the surfactant lining of the alveolar spaces. In line with these observations are the downstream effector proteins that have been identified in the GM-CSF signaling cascade of AM. Multiple studies highlighted the crucial role of the transcription factor PU.1 (SPI1) and further down the line the AM master transcription factor peroxisome proliferator-activated receptor gamma (PPARγ), which is also described in AM development and maturation [25,26]. PPARγ has implications in fatty acid metabolism and activates the transcription of ATP-binding cassette subfamily G member 1 (*ABCG1*), encoding a transmembrane lipid transporter that regulates the cholesterol efflux in macrophages [27,28]. Of note, recent insights into cholesterol metabolism of AM increased the understanding of the pathophysiology of PAP and, thus, opened new avenues for potential therapeutic implications [24].

In the context of secondary PAP, the exact pathophysiology is not elucidated yet, although it seems to be associated with decreased numbers or impaired functionality of AMs. A multitude of hematological and nonhematological diseases were reported to cause secondary PAP [13]. The most commonly reported hematological disorders have been chronic myeloid leukemia (CML) and myelodysplastic syndrome (MDS) [29], which lead to reduced numbers of functional monocytes that could substitute resident AMs in acute stress reactions. In line with this, acute myeloid leukemia (AML) or cutaneous T cell lymphoma have also been reported as cause a of secondary PAP [29]. Another group of diseases that can be associated with secondary PAP are immunodeficiencies. Here, thymic alymphoplasia [30], immunoglobulin A deficiency [31], GATA2 deficiency (MonoMac disease) [32] and adenosine deaminase-deficient severe combined immunodeficiency (ADA-SCID) [33] are inherited diseases that have been observed as drivers of secondary PAP. In these cases, the immune cell intrinsic deficiencies might explain the pathophysiology as also in this case monocytes as a pool for AM replenishment in acute lung stress responses might be inadequate or unavailable. However, also other conditions that lead to immunosuppression can cause secondary PAP, like the acquired immunodeficiency syndrome (AIDS) that results from HIV infection [34] or drug-induced immunosuppression for example by corticosteroids [35], busulfan [36], mycophenolate mofetil [37], and leflunamide [38] during treatment of autoimmune diseases or after solid organ transplantation (described, for example, for lung and kidney transplantation) [39,40] as well as hematopoietic stem cell transplantation (HSCT) [41,42]. Besides immunosuppression, chronic inflammation or infections can also be linked to secondary PAP, as described for infections with Cytomegalovirus, *Mycobacterium* (*M*.) *tuberculosis*, *Nocardia*, or *Pneumocystis carinii* [43,44,45,46]. Here, exhaustion of the AM pool and insufficient replenishment by monocytes due to continuous stress reactions might explain the pathophysiological mode of action. While explanations for hematological disorders might seem rather obvious, the associations with nonhematological causes of secondary PAP are more complicated. Here, a variety of different diseases have been described in association with secondary PAP, like the systemic vasculitis Behçet syndrome [47], systemic lupus erythematosus [48], Wegener’s granulomatosis [37], lysinuric protein intolerance [49], *OAS1* [50] and *MARS* mutations [51]. A special case is also inhalation of chemicals or dust as a driver of PAP. Various agents have been reported, such as cigarette smoke, silica, aluminum, cellulose fibers, titanium dioxide, indium-tin oxide and others [13]. However, although the inhalation of chemicals might likely harm AM and therefore cause PAP, the causal relationship between exposure and disease onset is not clear and it is debated whether this pathology can be classified as secondary PAP or rather causes autoimmune PAP [52,53].

#### Therapeutic Approaches for PAP

Streamlined therapy depends always on the form and/or clinical presentation of PAP. For a long time, the treatment of hereditary PAP was rather limited to symptomatic treatment only. The standard first line therapy since the 1960s has been repetitive whole lung lavage (WLL), an invasive procedure that requires general anesthesia and is linked to cardiovascular morbidity [13]. This mechanical removal of the accumulated surfactant material during WLL relieves symptoms for some time, although the effectiveness and the duration of the effect vary a lot among individual patients. As surfactants are continuously produced in the lungs, new material will accumulate and so many patients need to undergo the procedure regularly. Other attempts to treat severe cases of PAP, where WLL has failed and fibrotic remodeling of the lung has occurred, include lung transplantation. Surprisingly, several studies reported that after successful engraftment of the donor lung, PAP symptoms recurred after some/several months [40,54,55,56]. Interestingly, donor AM were absent in these patients and had been replaced by defective host monocyte-derived AM, further highlighting the crucial role of AM in the disease pathophysiology of PAP. In parallel to the understanding of the pathophysiology of PAP, new treatment strategies have evolved over time that try to tackle the cause of the disease. It became clear that AM are the main players in this game and that the endogenous AM pool can—in defined situations—be replaced by monocyte-derived macrophages as for example shown post irradiation. Thus, HSCT appeared to be a promising treatment option for PAP. Studies in a murine *Csf2rb^−/−^* model confirmed that allogeneic and autologous HSCT in combination with gene therapy can efficiently cure the PAP disease phenotype [57,58,59] (see also Table 1). Although these murine proof of concept studies showed promising results, clinical translation is hampered as many patients suffer from severe comorbidities, for example due to lung infections, and are in a poor general condition that is (often) not compatible with required preconditioning prior to HSCT. One PAP patient even died due to an overwhelming infection after preconditioning [60]. However, with improvements in the preconditioning regimens, HSCT has become available for more patients and recently, successful use of HSCT was reported in secondary [61,62] and hereditary [63] PAP patients, and indeed cured these patients. Another attempt to replace the malfunctional AM without severe preconditioning is the direct administration of macrophages to the lungs without the detour through the bone marrow niche. Here, macrophages from different stem cell sources (HSCs and induced pluripotent stem cells (iPSCs)) and species (mouse, human) were used and several studies reported significant improvement of disease parameters in *Csf2rb^−/−^*, *Csf2ra^−/−^*, as well as human IL-5/GM-CSF knock-in PAP mice after pulmonary macrophage transplantation (PMT) [22,64,65,66,67,68,69]. Irrespective of the origin, the administered macrophages engrafted in the lung niche and adapted a monocyte-derived AM-like phenotype that was reflected by surface marker profile and transcriptome.

Besides cell-based therapy options, pharmacologic therapeutics also emerged with growing knowledge in disease pathophysiology. Given the pivotal role of GM-CSF signaling in PAP, GM-CSF supplementation can be a valid therapeutic option for autoimmune PAP patients when enough GM-CSF is supplied to outperform the neutralizing antibodies [13]. Initial treatments in patients were performed with subcutaneous injection. However, the response of the patients was mixed and no larger clinical trials have been performed to confirm clinical relevance. The first attempts for topical application of GM-CSF have been made in *Csf2^−/−^* mice, in which inhaled GM-CSF significantly decreased phosphatidylcholine and surfactant protein B levels and normalized lung histology and AM maturation [70]. Several studies also reported benefit of aerosolized GM-CSF treatment in autoimmune PAP patients, thus representing a promising therapy option in autoimmune PAP [71]. However, GM-CSF augmentation therapy is not a therapeutic option for hereditary PAP caused by *CSF2R* mutations. As mentioned earlier, the PU.1-PPARγ-ABCG1 axis downstream of the CSF2R is of high relevance in the development and function of AMs. Recently, small molecules that act on this axis were investigated as potential drugs to treat PAP. Pioglitazone, a PPARγ agonist, was shown to restore cholesterol clearance of *Csf2^−/−^* and *Csf2rb^−/−^* AMs in vitro and in vivo and thereby decrease PAP disease severity [24]. This study also highlighted the tremendous role of cholesterol metabolism in PAP pathophysiology that was up to then underestimated. Likewise, a Liver X Receptor (LXRα) agonist that was discussed as a potential therapeutic in atherosclerosis as it influences the expression of *Abca1* and *Abcg1* and thus, cholesterol efflux, proved to decrease cholesterol accumulation in murine *Csf2^−/−^* macrophages previously exposed to PAP surfactant [24]. While there is no LXRα agonist that can safely be applied in humans, clinical translation might be challenging. In contrast, pioglitazone has already been administered to PAP patients in an ongoing clinical trial (NCT03231033) [72]. Another drug involved in cholesterol metabolism that was investigated in the context of PAP is statin. A study by McCarthy et al. presented increased cholesterol efflux in *Csf2rb^−/−^* AMs ex vivo, decreased cholesterol accumulation and amelioration of PAP in vivo and finally also therapeutic benefit and improved radiological findings in PAP patients under statin therapy [73]. Given the constant development and progress in research our knowledge of AMs and PAP will continuously grow and open up new cause-directed therapeutic options.

**Table 1:** Emerging macrophage-targeted therapies.

		Therapeutic Intervention								
	Disease	Cells	Drugs	Type of Cells	Type of Drug	Administration	Pre-Clinical Model	Human Studies	References	
**Surfactant Homeostasis/GM-CSF Pathway**	Pulmonary Alveolar Proteinosis (PAP) CSF2RB deficiency	x		Murine HSCs		i.v.	mouse		Nishinakamura et al 1996, Kleff et al. 2008, Hetzel et al. 2019	[57,58,59]
	Pulmonary Alveolar Proteinosis (PAP) secondary PAP and GATA2 deficiency	x		Human HSCs		i.v.		x	Tanaka-Kubota et al. 2018, van Lier et al. 2020, Ozcelik et al. 2020	[61,62,63]
	Pulmonary Alveolar Proteinosis (PAP) CSF2RA/CSF2RB deficiency	x		murine HSC-derived Macrophages		i.t.	mouse		Suzuki et al. 2014, Happle et al. 2014	[64,66]
	Pulmonary Alveolar Proteinosis (PAP) CSF2RA deficiency	x		human HSC-derived Mac		i.t.	mouse		Happle et al. 2014	[66]
	Pulmonary Alveolar Proteinosis (PAP) CSF2RB deficiency	x		murine iPSC-derived Macrophages		i.t.	mouse		Mucci et al. 2018	[65]
	Pulmonary Alveolar Proteinosis (PAP) CSF2RA deficiency	x		human iPSC-derived Macrophages		i.t.	mouse		Happle et al. 2018	[67]
	Pulmonary Alveolar Proteinosis (PAP) GM-CSF deficiency		x		GM-CSF	aerosole inhalation	mouse		Reed et al. 1999	[70]
	Autoimmune PAP		x		GM-CSF	inhaled and s.c.		x	Sheng et al. 2018	[71]
	Pulmonary Alveolar Proteinosis (PAP) GM-CSF and CSF2RbB deficiency		x		Pioglitazone (PPARy agonist)	oral	mouse		Sallese et al. 2001	[24]
	Pulmonary Alveolar Proteinosis (PAP) GM-CSF and CSF2RB deficiency		x		Liver X Receptor agonist	oral	mouse		Sallese et al. 2001	[24]
**Infections**	*Pseudomonas aeruginosa* infection	x		human iPSC-derived macrophages		i.t.	mouse		Ackermann et al. 2018	[74]
	*Francisella tularensis* infection		x		mannosylated ciprofloxacin polymeric prodrugs	i.t.	mouse		Chen et al. 2018, Su et al. 2018	[75,76]
	*Burkholderia pseudomallei* pulmonary meloidosis		x		macrophage-targeted polyciprofloxacin prodrug	i.t. aerosolization			Chavas et al. 2021	[77]
	Cystic fibrosis (CF) associated pulmonary *Pseudomonas aeruginosa* infection	x		murine HSCs		i.v.	mouse		Brinkert et al. 2020	[78]
	MSMD associated pulmonary BCG infection	x		murine HSCs		i.v.	mouse		Hetzel at al. 2018	[79]
**Imbalanced Pulmonary Immunity**	Allergic asthma		x		Omalizumab (Anti-IgE-AB)	s.c.		x	Humbert et al. 2018	[80]
	Allergic asthma		x		CCR2-05 (anti-CCR2 AB)	i.v.	monkey		Mellado et al. 2008	[81]
	Allergic asthma	x		murine alveolar macrophages		i.t.	rat		Careau et al. 2004	[82]
	Idiopathic pulmonary fibrosis		x		FA-TLR7-54 (folate-targeted TLR7 agonist)	i.v.	mouse		Zhang et al. 2020	[83]
	Idiopathic pulmonary fibrosis		x		RP-832c (CD206 targeting peptide)	s.c.	mouse		Ghebremedhin et al. 2021	[84]
	Idiopathic pulmonary fibrosis		x		liposomal siRNA against spliceosome associated factor 1 (*Sart1*)	i.t.	mouse		Pan et al. 2021	[85]
	Idiopathic pulmonary fibrosis		x		AA6216 (phosphodiesterase 4 (PDE4) inhibitor)	oral	mouse		Matsuhira et al. 2020	[86]

Abbreviations: Hematopoietic Stem Cell (HSC); intra-venously (i.v.); intra-tracheally (i.t.), sub cutanous (s.c.), Induced pluripotent stem cell (iPSC), Toll like receotpr (TLR), peroxisome proliferator-activated receptors (PPA).

### 2.2. The Role of AM in Infections of the Lower Respiratory Tract

Besides their eminent role in surfactant homeostasis, AM are also indispensable for the prevention of infections. Given their high abundance and their prominent location in the alveoli where they encounter pathogens and dusts that are transported into the lungs every day, it is not surprising that AM are the most important cell type in the recognition of pathogens and the initiation of pulmonary immune responses. Being constantly exposed to a multitude of stimuli, AM have to finely balance their response. On the one hand, they need to dampen the immune response to prevent tissue damage due to overwhelming inflammation, but on the other hand, they need to appropriately react to harmful, invading pathogens to prevent severe lung infections. This narrow ridge is indeed a balancing act; despite long-held beliefs that the lung is not sterile in steady state, it in fact contains a resident microbiome that also comprises potentially harmful pathogens [87,88]. In their role as immune sentinels and first-line defense against pathogens, AM apply different strategies to cope with pathogen invasions. In contrast to the adaptive immunity, AM recognize a broad range of pathogens via pattern recognition receptors such as Fcy-receptors, Toll-like receptors or C type lectin receptors leading to an activation of inflammatory pathways by the activation of, e.g., NFκB and the initiation of the immune responses [89]. Importantly, these different receptor families are often activated simultaneously to induce an adequate immune response [90]. Besides the production of proinflammatory cytokines and chemokines that attract and activate other immune cells, a major task of AM is the phagocytosis and degradation of bacteria, as evident from their name “big eaters”. After the uptake, bacteria either become degraded in the phagolysosome or, if the intracellular burden exceeds the lysosomal killing capacity, AM undergo apoptosis to kill the intracellular pathogens, e.g., as described for *Streptococcus (S.) pneumoniae* [91,92].

Over time, pathogens developed a number of different strategies to evade the immune response of macrophages. Some pathogens for example employ molecular mimicry by expressing host sialylated glycans, which bind to immunosuppressive sialic acid-binding immunoglobulinlike lectins (Siglecs) on innate immune cells and prevent the activation of these cells [93,94]. Additionally, the production of various virulence factors and toxins is utilized by the pathogens to evade immune responses and eradicate host immune cells. In this context, *Staphylococcus (S.) aureus*, for example, has been described to secrete several toxins, such as α-toxin or leukotoxins, that efficiently damage the host cell membrane by forming β-barrel pores in the cytoplasmic membrane leading to cell leakage and finally cell lysis [95]. Moreover, many (intracellular) pathogens like *S. aureus*, *M. tuberculosis*, *Legionella pneumophila*, *Bacillus anthracis* and others [96,97] even use AM as a reservoir in which they can persist and reproduce. Other evasion strategies make use of the ambiguity of AM between proinflammatory pathogen defense and anti-inflammatory tissue protection. To avoid intracellular killing by, e.g., nitric oxide (NO), pathogen prefer the anti-inflammatory M2 phenotypes of macrophages. Some pathogens like *Chlamydia pneumoniae* or *M. tuberculosis* even actively drive AM towards the M2 phenotype to better suite their requirements. For example, *M. tuberculosis* activates the expression of *PPARγ* to polarize AM towards the M2 phenotype and, in addition, benefits from the metabolic changes in AM, as it can use the produced lipids as nutrients [98,99]. However, bacteria can also highjack other metabolic pathways to their advantage, for example the host iron or zinc metabolism [100,101]. The M2 phenotype is also beneficial for the pathogen, as it dampens the overall immune response and creates a favorable environment in the whole lung niche. Anti-inflammatory prostaglandin E2 (PGE_2_) can be induced by *M. tuberculosis* to prevent bacterial killing by NADPH oxidase and, similarly, the influenza virus has been described to induce PGE_2_ to suppress type 1 interferon (IFN) response, which is crucial for antiviral responses. On the other hand, type 1 IFN responses induced by viral infection can lead to a favorable environment for other pathogens, leading to bacterial superinfection and pneumonia after viral infection [102,103,104,105]. In addition to the alteration of the cytokine response, the decrease in AM numbers during influenza A infection also enables bacterial or fungal superinfection [106,107].

Tissue resident AM and also monocyte-derived AM surviving infections can show changes in their phenotype and function. In this line, it has been shown that AM remaining after an infection are in an immune-paralyzed, tolerogenic state that renders them susceptible to secondary infections [108,109]. A recent study by Roquilly and colleagues demonstrated that the AM present decreased phagocytic capacity for several months after severe pneumonia, which is mediated by the immunosuppressive environment after infection and here, in particular, SIRPα is a key player in mediating tolerance in AM [108]. Although the nomenclature on this topic is still under debate and this immune-comprised state is referred to as “trained immunity”, “immune paralysis” or “innate imprinting” in the field, it seems rather clear that certain environmental cues create an immunosuppressive milieu after infection that alters innate immunity by inducing long-lasting epigenetic changes. Anti-inflammatory signaling pathways like IL-10, TGFβ, CD200, and prostaglandins seem to play major roles in mediating susceptibility to secondary infections due to non/low-responsive innate immunity [109]. However, priming of innate immune cells is not necessarily a disadvantage. Species that lack adaptive immunity (like plants or invertebrates) also show enhanced functionality of the innate immune cells after initial priming [110,111]. In parallel to the negative effects described before, the beneficial effects of trained immunity, like heightened and quicker response upon re-exposure to a certain pathogen, are also mediated by epigenetic changes. Here, NOD2 and dectin-1 signaling have been identified to induce stable changes in H3K4 histone trimethylation [112]. Whether the priming of AM has a beneficial effect or not might be dependent on the type of pathogen they are encountering, the severity of the inflammation this pathogen is causing, the frequency of the encounters and also on the origin of the remaining AM (monocyte-derived vs. fetal liver monocyte-derived), which will be discussed later.

In addition to the already challenging tasks for AM, the situation becomes even more problematic when genetic disorders weaken the immune capabilities of AM. In this respect, defects in the previously highlighted IFN pathways have detrimental effects on AM host defense against various pathogens. Since 1996, 15 genes have been identified, which all have implications in the IFNγ/IL-12 axis leading to Mendelian susceptibility to mycobacterial disease (MSMD) [113]. MSMD is a genetically heterogenous but clinically distinct group of disorders that is characterized by the susceptibility only weakly virulent mycobacteria and attenuated *Bacillus Calmette-Guérin* (BCG) vaccine in otherwise healthy individuals. Mycobacterial infections in these patients are not limited to the lungs; however, *M. tuberculosis* as well as the environmental *Mycobacterium* (*M.*) *avium* can cause severe pulmonary infections and granuloma formation in these patients. Another group of individuals highly susceptible to severe pulmonary infections are cystic fibrosis (CF) patients. CF is caused by mutations in the cystic fibrosis transmembrane conductance regulator (*CFTR*) gene, which lead to impaired ion transport in a variety of different secretory epithelial cell types from the airways to the intestine [114]. In particular, the defective homeostasis of mucus clearance in the lung contributes to severe clinical phenotypes by impaired lung function. However, as chronic airway infections also represent one of the main causes of morbidity and mortality in CF patients, recent studies investigated the impact of *CFTR* mutations on the functionality of AM. Indeed, alveolar macrophages from murine *cftr^−/−^* models, show reduced phagosome acidification and bactericidal activity [78,115].

Recently, it was postulated that genetic predispositions in IFN-mediated immunity are also key to severe cases of coronavirus disease 2019 (COVID-19) caused by infection with severe acute respiratory syndrome coronavirus 2 (SARS-CoV-2) [116]. Loss-of-function mutations in Toll-like receptor 3 (TLR3) and interferon regulatory factor 7 (IRF7), which are also important in influenza infections, can lead to increased vulnerability to SARS-CoV-2 infections and might explain severe cases in young and otherwise healthy individuals [117]. Similarly, a genome-wide association study identified reduced interferon α receptor 2 (IFNAR2) expression as a risk factor for severe case progression [118]. Additionally, preformed autoantibodies against IFNω and IFNα were reported in 2.6% of women and 12.5% of men with severe COVID-19 pneumonia [119]. It was also reported that upon SARS-CoV-2 challenge, AM are not able to sense the virus and, in contrast to influenza A or Sendai virus infections, are not responding to IFN production [120]. On the other hand, type I IFNs might stimulate the expression of angiotensin-converting enzyme 2 (ACE2) [121], the entry receptor for SARS-CoV-2, which is probably expressed on AM [122] although conflicting reports exist on these facts [123,124]. Since AM are also the first immune cells in the lung to encounter SARS-CoV-2, it is likely that they serve as a reservoir for the virus. It was debated that patients with increased AM numbers could be more susceptible to severe COVID-19 progression [125], as increased AM numbers can be found in high-risk groups like chronic obstructive pulmonary disease (COPD) patients [126] and in the elderly. In fact, the numbers of murine AMs increase with age, but the AM functionality, like bactericidal capacity, decrease over time [99,127,128].

#### Therapeutic Approaches to Treat Pulmonary Infections

Since their discovery almost a century ago, antibiotics have been the standard of care for most bacterial infections. However, their systemic application is hampered by the insufficient penetration of deep tissue (especially in the lung) and also sometimes associated with severe and dose limiting side effects. Moreover, due to their imprudent use and unavoidable coevolutionary effects, the number of antibiotic resistant germs is steadily increasing [129]. Methicillin resistant *S. aureus* (MRSA) and other multiresistant pathogens are a major threat and their proportion of nosocomial infections are concerning [130]. As the development of new antibiotic agents has been neglected due to, e.g., economic reasons, innovative treatment strategies have to be developed to tackle the problems of pulmonary infections.

As mentioned before, AM represent the first line of cellular host defense in the lung, proficiently phagocytose bacteria and initiate and coordinate pulmonary immune responses. Therefore, several strategies aim to further support their function and overcome problems associated with systemic administration of antibiotics. In the past few years, a variety of different methods have evolved that allow for the local, pulmonary delivery of substances specifically to AM including for example nanoparticles or liposomes [131]. In the context of pulmonary infections, several reports have shown that macrophage-targeted antibiotics or predrugs can increase survival rates in murine pulmonary infection models and also target the intracellular pathogens that reside in AM. Here, in particular Ciprofloxacin and its derivates were exploited to prevent or treat *Francisella tularensis* infections [75,76] or *Burkholderia pseudomallei* pulmonary meloidosis [77]. Similar treatment approaches were also evaluated in the context of tuberculosis [132,133]. In addition to fortifying endogenous AM, another promising strategy to combat bacterial infections could be an immunotherapy approach employing the adoptive transfer of macrophages to the lungs. In proof-of-concept studies our group demonstrated that the transplantation of human iPSC-derived macrophages can rescue immunodeficient mice from acute *Pseudomonas* (*P.*) *aeruginosa* or *S. aureus* infections as demonstrated by the lower bacterial burden and reduced inflammation associated parameters in treated animals [74] (and own unpublished data). To further improve the potential of this cell therapy concept, an enhancement of the transplanted macrophages might additionally support innate immune reaction. Following this line of thought, the polarization of macrophages towards a proinflammatory M1 phenotype prior to transplantation, e.g., by stimulation with INFy, might further enhance the antimicrobial effect and improve the therapeutic outcome. As the functional status of macrophages in terms of polarization is reflected by the expression of specific surface molecules, the optimal treatment condition can be evaluated before transplantation.

For instance, patients who suffer from a genetic predisposition to infections due to malfunctional AM are in urgent need of advanced therapeutic strategies. While it is not yet clear if the previously discussed administration of macrophages or macrophage-targeting drug delivery platforms might result in a permanent cure for these patients, strategies such as HSCT could represent an option for long-lasting prevention of infection. It is well-known that irradiation and chemotherapy preconditioning before HSCT are also depleting the pulmonary AM niche, which can then be repopulated by donor-derived monocytes. An allogeneic HSCT approach transplanting healthy HSC into cystic fibrosis (CF) mice recently demonstrated prevention of pulmonary *P. aeruginosa* infection by replacing malfunctioning AM [78]. By combining autologous HSCT with gene therapy, we previously demonstrated protection against pulmonary BCG infection in *Ifnγr1*-deficient MSMD mice [79]. As a future perspective, gene therapeutic strategies could not only be used to correct defective genes but also to enhance the resistance and functionality of macrophages to fight infections.

### 2.3. Imbalanced Pulmonary Immunity in Asthma and Fibrosis

Besides their important role in initiation and coordination of immune responses, AM are also crucial players in terminating immune response after an infection is resolved, as well as in maintaining tissue homeostasis under steady state conditions. If this dual function becomes out of balance, an overshooting immune system can lead to tissue damage. A prominent example of a chronically inflamed lung is asthma, which has been increasing in prevalence over several decades. This disease is associated with an allergen-trigger response resulting in an eosinophil-predominant type 2 inflammation and subsequent airway remodeling and hyperresponsiveness. Patients typically present with shortness of breath, cough and chest tightness. In the past, much attention was paid to the underlying type 2 inflammation; however, several studies show an important influence of different macrophage populations on the development of asthma [134]. Until now, the role of AM in the development and maintenance of the inflammatory status leading to asthma remain elusive and several studies revealed different outcomes after the depletion of monocyte/macrophages, showing either beneficial or detrimental effects on the development of asthma. This discrepancy might be explained by the different roles of the embryonic resident AM, infiltrating monocytes and infiltrating monocyte-derived AM. Whereas several studies have shown that a depletion of monocytes in the peripheral blood can lead to an attenuation of allergic inflammation, a depletion of resident AM resulted in increased inflammatory signaling [135,136]. However, the picture is not so clear, as it has also been demonstrated that infections with a gammaherpes virus can lead to protection from allergic asthma and that this observation is caused by replacement of AM with regulatory monocytes [137]. In addition to the role of different monocyte/macrophage populations, asthma is also associated with altered microbial colonization of the airways and distinct microbial signatures. This might be caused by a defective phagocytotic potential of AM, but also peripheral blood monocytes, indicating asthma as a systemic disease [138,139]. Further studies demonstrate that in children the severity of asthma is associated with increased oxidative stress and reduced phagocytotic potential of AM [140]. Of note, the ex vivo treatment of AM with antioxidants could rescue the phagocytotic potential suggesting that targeting of the oxidant/antioxidant balance could open new therapeutic strategies [141].

As mentioned before, AM are also essential for wound healing and for the restoration of tissue integrity and homeostasis. The increased activation of AM in this repair process can cause tissue scarring and finally lead to pulmonary fibrosis. Similar to the situation in asthma, the role of monocyte-derived versus embryonic resident AM is not completely clear. However, in fibrosis most evidence hints towards a negative effect of infiltrating monocytes. Using scRNA sequencing, Aran and colleagues identified a profibrotic macrophage with a transitional gene expression profile resembling an intermediate state between monocyte-derived and resident AM that localized to the fibrotic niche and promoted fibrosis in a bleomycin-induced mouse model [142]. The trafficking of monocytes to the lungs occurs along a CCL2/CCR2 axis, in which IL-1R-associated kinase M (IRAK-M) has been identified as key molecule for CCR2 upregulation after bleomycin-induced lung injury leading to increased monocyte infiltration [143]. In the lungs, the immunoreceptor CD300c2 is crucial for AM activation and secretion of chemoattractants that cause neutrophil invasion and inflammation in bleomycin-induced fibrosis [144]. Additionally, Notch signaling via recombination signal-binding protein Jκ (RBP-J) regulates monocyte recruitment and monocyte-derived AM activation as well as TGF-β secretion and thus, contributes to fibrosis progression [145]. Other studies also supported the idea that profibrotic macrophages are mainly monocyte-derived as clodronate depletion of resident AM before bleomycin treatment also led to fibrosis [145,146]. The profibrotic gene transcription profile of the recruited monocytes vanished over time and the cells more and more resembled the resident AM profile, indicating that the tissue niche derived imprinting is stronger than the endogenous inflammatory monocyte gene signature [146,147]. Profibrotic macrophages and the upregulation of human ortholog genes have also been identified in fibrosis patients [148]. Profibrotic actions of AM include secretion of factors like TGF-β, IL-1β, CCL18, and PDGF that activate fibroblasts, which in turn secrete extracellular matrix components and, thus, cause fibrosis [149]. However, similar to observations in asthma, defects in phagocytosis have also been described in fibrosis. Profibrotic macrophages from patients presented an immature phenotype and impaired phagocytosis [2,148,150]. As efferocytosis of apoptotic cells is a key feature of AM in maintaining homeostasis in the lung tissue, it is not surprising that defects in apoptotic cell clearance drive inflammatory processes and participate in the pathogenesis of fibrosis. Additionally, defects in phagocytosis and efferocytosis have also been implicated in other lung diseases with AM as crucial cells during pathogenesis, e.g., in chronic obstructive pulmonary disease (COPD) [151].

Taken together, although several studies demonstrate an important impact of AM on asthma and fibrosis, further research is needed to shed light on the beneficial or harmful role of macrophages of different origins and phenotypes at different stages of these diseases.

#### Therapeutic Approaches Tackling Asthma and Pulmonary Fibrosis

As asthma is characterized by airway hyperresponsiveness and a shortage of breath, a common symptomatic therapy is the short-term or long-term administration of ß-2-agonists as bronchodilators. Furthermore, in order to address the underlying chronic inflammation, asthma patients are also treated by immunosuppressive glucocorticoids, e.g., cortisone. These drugs target multiple cell types, including epithelial and immune cells, but especially macrophages [138]. In AM, corticoid treatment results in the epigenetic remodeling of promotor sequences by histone deacetylases and the suppression of proinflammatory genes [152], resulting in reduced levels of for example IL1b, TNFa, PGE2 and leukotriene B4 production. Moreover, this treatment increases anti-inflammatory IL-10 production and reduces oxidative stress in AM. However, patients with severe asthma often respond poorly to corticosteroids, which could be associated with a resistance of AM to respond to corticosteroids and a skewing towards a dominant M1 phenotype, probably driven by an aberrant p38 pathway activity [138]. As an alternative way to reduce the inflammation underlying asthma, several specific targeted therapies against inflammation cytokines have also been studied. A prominent example is an anti-IgE antibody (Omalizumab) [80], which inhibits the binding of IgE to mast cells and eosinophils and furthermore reduces the production of IgE by B cells [153]. In addition, several other antibodies targeting the IL-4 and IL-5 pathways have been investigated [154].

Given the important role of different monocyte/macrophage populations in different disease stages of asthma, targeted therapies directly depleting or replacing this cell populations have also been evaluated in animal models. As mentioned previously, infiltrating monocytes and monocyte-derived AM in the early adaptation stages contribute to the progression of inflammation and the development of asthma, suggesting selective inhibition of monocyte recruitment as a potential therapeutic approach. Indeed, Mellado and colleagues demonstrate that blocking the recruitment of inflammatory monocytes by the systemic administration of an anti-CCR2 antagonist (CCR2-05) monoclonal antibody in a cynomolgus monkey model delayed allergic reactions and reduced the number of inflammatory cells in the lung, highlighting the utility of this approach for early stages of asthma [81]. Another study focused on the important anti-inflammatory and beneficial role of resident AM in asthma. The authors transplanted AM derived from an allergy-resistant rat model into the lungs of an ovalbumin-sensitized allergy rat model, after removing the endogenous AM population by the application of clodronate liposomes. Following the exchange of AM, airway hyperresponsiveness in transplanted animals was reduced to WT levels [82]. These data again highlight the important anti-inflammatory role of AM in asthma and furthermore suggest that the transplantation of macrophages, after maturation and polarization into an appropriate phenotype in vitro, could represent a therapeutic option for patients suffering from severe asthma.

In contrast to asthma, therapeutic options for pulmonary fibrosis are very limited and mainly constitute symptomatic therapy like oxygen supply. The only FDA-approved pharmaceutics are nintedanib and pirfenidone, which only slow down fibrosis progression but are not able to reverse existing fibrotic changes [155]. In severe cases, lung transplantation remains the only option. Modern approaches try to tackle different crucial factors in the pathogenesis of fibrosis, which include counteracting the activated profibrotic macrophages and secreted profibrotic factors. The specific activation status of these macrophages, which is reflected by the surface marker profile, can be used to specifically track and modify the macrophages. The in vivo reprogramming of profibrotic macrophages that are main contributors to disease progression appears to be a promising approach. In this line, a Toll-like receptor 7 (TLR7) agonist was targeted to macrophages via folate to avoid systemic toxicity. In a bleomycin-induced fibrosis mouse model, treatment with this compound resulted in reprogramming of profibrotic M2 macrophages to antifibrotic macrophages with significant changes in the cytokine secretion profile as well as a significant decrease of collagen deposition [83]. The fact that folate receptor β is mainly expressed on activated infiltrating monocytes and not on embryonic resident AM supports the fact that monocyte-derived AM are mainly responsible for fibrosis progression. Another approach to reprogram profibrotic macrophages recently reported in a preprint is the use of the host defense peptide RP-832c that selectively targets CD206 on M2 macrophages [84]. The authors report a dose-dependent decrease of CD206, TGF-β and α-SMA expression in bleomycin-induced fibrosis. Another approach reported liposome-mediated delivery of siRNA against spliceosome associated factor 1 (*Sart1*), which was shown to be involved in oxygen-independent HIF-1α degradation and macrophage M2 polarization, to attenuate M2 macrophage polarization in fibrotic lungs [85]. Additionally, a novel phosphodiesterase 4 (PDE4) inhibitor has been tested for antifibrotic effects in a bleomycin-induced mouse model [86]. While a significant decrease in macrophage activation and secretion of profibrotic TGF-β and TNF-α was reported, the dose-limiting toxicity of other PDE4 inhibitors was not excluded in this approach. Taken together, the reprogramming of profibrotic macrophages is a promising approach for the treatment of fibrosis. In this context, the testing of new immune modulatory compounds as well as specifically targeting known compounds to AM could pave new ways for fibrosis therapy.

## 3. Future Perspectives

Given the important roles of AM in surfactant homeostasis, pathogen clearance, immune activation and regulation, several therapeutic strategies directly targeting absent or malfunctional AM have been developed in order to provide novel therapeutic options for severe diseases (see also Table 1). Besides the possibility to modulate the development, functionality or metabolism of AM by specific drugs, cell-replacement and immunotherapy strategies are also emerging. Irrespective of the origin, multiple subsets of monocytes/macrophages such as yolk sac macrophages, fetal liver-, or adult bone marrow-monocytes have the ability to engraft in the lung. In a pioneering study, van de Laar and colleagues demonstrated that fetal liver-derived macrophages outcompeted other types of macrophages, i.e., yolk sac macrophages, upon competitive administration into the lungs [27]. However, all types of macrophages were able to engraft and adapt to the niche, again highlighting the pivotal role of niche-specific instructive signals. Given the longevity of AM, which are able to maintain their population under steady state conditions, cell replacement strategies employing monocyte or stem cell-derived macrophages promise a long-term and causative treatment option for several diseases, with PAP being the most prominent example.

However, macrophage-based therapies require the availability of autologous or allogenic donor monocytes. While it remains challenging to derive these cells in suitable quantities and qualities and there is also a high donor to donor variability, iPSC, with their unlimited proliferation and differentiation potential in vitro, offer an interesting alternative cell source. Several studies have demonstrated the efficient generation of iPSC-derived macrophages and recent studies further developed this technique for scalable production in industry-compatible stirred tank bioreactors [74]. Moreover, macrophages from pluripotent stem cells (PSC), similar to TRM, seem to follow a primitive developmental program, which is independent of MYB, but dependent on RUNx1- and SPI1 (PU.1) [156]. In fact, macrophages derived from iPSC have been used to study a plethora of macrophage related disorders and have also been used to treat PAP, demonstrating the long-lasting effects and durability of transplanted cells post-intrapulmonary transfer [65,67,157]. To further highlight the attractiveness of these cells, transplanted iPSC-macrophages also converted into an AM-like phenotype [67], fostering the use of these cells for future regenerative therapies. Here, the scalable production of macrophages from iPSC, in combination with prestimulation towards context-dependent pro- or anti-inflammatory phenotypes and genetic enhancement of cells, e.g., by CRISPR/Cas9 technology opens new avenues for upcoming macrophage-based therapies.

## Data Availability

Not Applicable.

## References

[1] Kulikauskaite J., Wack A. (2020). Teaching Old Dogs New Tricks? The Plasticity of Lung Alveolar Macrophage Subsets. Trends Immunol..

[2] Shi T., Denney L., An H., Ho L., Zheng Y. (2020). Alveolar and lung interstitial macrophages: Definitions, functions, and roles in lung fibrosis. J. Leukoc. Biol..

[3] Mass E., Ballesteros I., Farlik M., Halbritter F., Günther P., Crozet L., Jacome-Galarza C.E., Händler K., Klughammer J., Kobayashi Y. (2016). Specification of tissue-resident macrophages during organogenesis. Science.

[4] Bian Z., Gong Y., Huang T., Lee C.Z.W., Bian L., Bai Z., Shi H., Zeng Y., Liu C., He J. (2020). Deciphering human macrophage development at single-cell resolution. Nat. Cell Biol..

[5] Evren E., Ringqvist E., Tripathi K.P., Sleiers N., Rives I.C., Alisjahbana A., Gao Y., Sarhan D., Halle T., Sorini C. (2021). Distinct developmental pathways from blood monocytes generate human lung macrophage diversity. Immunity.

[6] Trapnell B.C., Whitsett J.A. (2002). Gm-CSF regulates pulmonary surfactant homeostasis and alveolar macro-phage-mediated innate host defense. Annu. Rev. Physiol..

[7] Bustamante J., Boisson-Dupuis S., Abel L., Casanova J.L. (2014). Mendelian susceptibility to mycobacterial disease: Genetic, immunological, and clinical features of inborn errors of IFN-gamma immunity. Semin. Immunol..

[8] Medeiros A.I., Serezani C.H., Lee S.P., Peters-Golden M. (2009). Efferocytosis impairs pulmonary macrophage and lung antibacterial function via PGE2/EP2 signaling. J. Exp. Med..

[9] Chen Y.-W., Huang M.-Z., Chen C.-L., Kuo C.-Y., Yang C.-Y., Chiang-Ni C., Chen Y.-Y.M., Hsieh C.-M., Wu H.-Y., Kuo M.-L. (2020). PM2.5 impairs macrophage functions to exacerbate pneumococcus-induced pulmonary pathogenesis. Part. Fibre Toxicol..

[10] Gonzalez Y., Carranza C., Iñiguez M., Torres M., Quintana R., Osornio A., Gardner C., Sarkar S., Schwander S. (2018). Effect of inhaled air pollution particulate matter in alveolar macrophages on local pro-inflammatory cytokine and peripheral interferon γ production in response to Mycobacterium tuberculosis. Lancet Glob. Health.

[11] Zhao H., Li W., Gao Y., Li J., Wang H. (2014). Exposure to particular matter increases susceptibility to respiratory Staphylococcus aureus infection in rats via reducing pulmonary natural killer cells. Toxicology.

[12] Sawyer K., Mundandhara S., Ghio A.J., Madden M.C. (2009). The Effects of Ambient Particulate Matter on Human Alveolar Macrophage Oxidative and Inflammatory Responses. J. Toxicol. Environ. Health Part A.

[13] Trapnell B.C., Nakata K., Bonella F., Campo I., Griese M., Hamilton J., Wang T., Morgan C., Cottin V., McCarthy C. (2019). Pulmonary alveolar proteinosis. Nat. Rev. Dis. Primers..

[14] Guilliams M., De Kleer I., Henri S., Post S., Vanhoutte L., De Prijck S., Deswarte K., Malissen B., Hammad H., Lambrecht B.N. (2013). Alveolar macrophages develop from fetal monocytes that differentiate into long-lived cells in the first week of life via GM-CSF. J. Exp. Med..

[15] Sakagami T., Beck D., Uchida K., Suzuki T., Carey B.C., Nakata K., Keller G., Wood R.E., Wert S.E., Ikegami M. (2010). Patient-derived Granulocyte/Macrophage Colony–Stimulating Factor Autoantibodies Reproduce Pulmonary Alveolar Proteinosis in Nonhuman Primates. Am. J. Respir. Crit. Care Med..

[16] Stanley E., Lieschke G.J., Grail D., Metcalf D., Hodgson G., Gall J.A., Maher D.W., Cebon J., Sinickas V., Dunn A.R. (1994). Granulocyte/macrophage colony-stimulating factor-deficient mice show no major perturbation of hematopoiesis but develop a characteristic pulmonary pathology. Proc. Natl. Acad. Sci. USA.

[17] Miyajima A. (1992). Molecular structure of the IL-3, GM-CSF and IL-5 receptors. Stem Cells.

[18] Suzuki T., Sakagami T., Rubin B.K., Nogee L.M., Wood R.E., Zimmerman S.L., Smolarek T., Dishop M.K., Wert S.E., Whitsett J.A. (2008). Familial pulmonary alveolar proteinosis caused by mutations in CSF2RA. J. Exp. Med..

[19] Suzuki T., Maranda B., Sakagami T., Catellier P., Couture C.-Y., Carey B.C., Chalk C., Trapnell B.C. (2010). Hereditary pulmonary alveolar proteinosis caused by recessive CSF2RB mutations. Eur. Respir. J..

[20] Tanaka T., Motoi N., Tsuchihashi Y., Tazawa R., Kaneko C., Nei T., Yamamoto T., Hayashi T., Tagawa T., Nagayasu T. (2011). Adult-onset hereditary pulmonary alveolar proteinosis caused by a sin-gle-base deletion in CSF2RB. J. Med. Genet..

[21] Nishinakamura R., Nakayama N., Hirabayashi Y., Inoue T., Aud D., Mcneil T., Azuma S., Yoshida S., Toyoda Y., Aral K.I. (1995). Mice deficient for the IL-3/GM-CSF/IL-5 beta c receptor exhibit lung pathology and impaired immune response, while beta IL3 receptor-deficient mice are normal. Immunity.

[22] Arumugam P., Suzuki T., Shima K., McCarthy C., Sallese A., Wessendarp M., Ma Y., Meyer J., Black D., Chalk C. (2019). Long-Term Safety and Efficacy of Gene-Pulmonary Macrophage Transplantation Therapy of PAP in Csf2ra−/− Mice. Mol. Ther..

[23] Suzuki T., Sakagami T., Young L.R., Carey B.C., Wood R.E., Luisetti M., Wert S.E., Rubin B.K., Kevill K., Chalk C. (2010). Hereditary pulmonary alveolar proteinosis: Pathogenesis, presentation, diagnosis, and therapy. Am. J. Respir. Crit. Care Med..

[24] Sallese A., Suzuki T., McCarthy C., Bridges J., Filuta A., Arumugam P., Shima K., Ma Y., Wessendarp M., Black D. (2017). Targeting cholesterol homeostasis in lung diseases. Sci. Rep..

[25] Shibata Y., Berclaz P.Y., Chroneos Z.C., Yoshida M., Whitsett J.A., Trapnell B.C. (2001). GM-CSF regulates alveolar macro-phage differentiation and innate immunity in the lung through PU. Immunity.

[26] Schneider C., Nobs S.P., Kurrer M., Rehrauer H., Thiele C., Kopf M. (2014). Induction of the nuclear receptor PPAR-gamma by the cytokine GM-CSF is critical for the differentiation of fetal monocytes into alveolar macrophages. Nat. Immunol..

[27] Baker A.D., Malur A., Barna B.P., Kavuru M.S., Malur A.G., Thomassen M.J. (2010). PPARγ regulates the expression of cholesterol metabolism genes in alveolar macrophages. Biochem. Biophys. Res. Commun..

[28] Thomassen M.J., Barna B.P., Malur A.G., Bonfield T.L., Farver C.F., Malur A., Dalrymple H., Kavuru M.S., Febbraio M. (2007). ABCG1 is deficient in alveolar macrophages of GM-CSF knockout mice and patients with pulmonary alveolar proteinosis. J. Lipid Res..

[29] Chaulagain C.P., Pilichowska M., Brinckerhoff L., Tabba M., Erban J.K. (2014). Secondary pulmonary alveolar proteinosis in hematologic malignancies. Hematol. Stem Cell Ther..

[30] Haworth J.C., Hoogstraten J., Taylor H. (1967). Thymic alymphoplasia. Arch. Dis. Child..

[31] Webster J.R., Battifora H., Furey C., Harrison R.A., Shapiro B. (1980). Pulmonary alveolar proteinosis in two siblings with decreased immunoglobulin A. Am. J. Med..

[32] Griese M., Zarbock R., Costabel U., Hildebrandt J., Theegarten D., Albert M., Thiel A., Schams A., Lange J., Krenke K. (2015). GATA2 deficiency in children and adults with severe pulmonary alveolar proteinosis and hematologic disorders. BMC Pulm. Med..

[33] Grunebaum E., Cutz E., Roifman C.M. (2012). Pulmonary alveolar proteinosis in patients with adenosine deaminase deficiency. J. Allergy Clin. Immunol..

[34] Ruben F.L., Talamo T.S. (1986). Secondary pulmonary alveolar proteinosis occurring in two patients with acquired immune deficiency syndrome. Am. J. Med..

[35] Hosoda C., Saito K., Fujimoto S., Yamanaka Y., Watanabe N., Miyagawa H., Kurita Y., Seki Y., Kinoshita A., Endo Y. (2019). Pulmonary alveolar proteinosis developing during steroid treatment in a patient with organizing pneumonia in association with atypical chronic myeloid leukemia. Clin. Case Rep..

[36] Watanabe K., Sueishi K., Tanaka K., Nagata N., Hirose N., Shigematsu N., Miake S., Yoshida M. (1990). Pulmonary Alveolar Proteinosis and Disseminated Atypical Mycobacteriosis in a Patient with Busulfan Lung. Pathol. Int..

[37] Shah S.K., Phan N.B., Goyal G., Sharma G. (2010). Pulmonary Alveolar Proteinosis in a 67-Year-Old Woman with Wegener’s Granulomatosis. J. Gen. Intern. Med..

[38] Wardwell N.R., Miller R., Ware L.B. (2006). Pulmonary alveolar proteinosis associated with a disease-modifying antirheumatoid arthritis drug. Respirology.

[39] Kadikoy H., Paolini M., Achkar K., Suki W., Gaber A.O., Anwar N., Jeroudi A., Barrios R., Abdellatif A. (2010). Pulmonary alveolar proteinosis in a kidney transplant: A rare complication of sirolimus. Nephrol. Dial. Transplant..

[40] Philippot Q., Cazes A., Borie R., Debray M.-P., Danel C., Nedelec M.H., Boudjemaa S., Sroussi D., Dupin C., Mal H. (2017). Secondary pulmonary alveolar proteinosis after lung transplantation: A single-centre series. Eur. Respir. J..

[41] Tomonari A., Shirafuji N., Iseki T., Ooi J., Nagayama H., Masunaga A., Tojo A., Tani K., Asano S. (2002). Acquired pulmonary alveolar proteinosis after umbilical cord blood transplantation for acute myeloid leukemia. Am. J. Hematol..

[42] Pidala J., Khalil F., Fernandez H. (2011). Pulmonary alveolar proteinosis following allogeneic hematopoietic cell transplantation. Bone Marrow Transplant..

[43] Witty L.A., Tapson V.F., Piantadosi C.A. (1994). Isolation of Mycobacteria in Patients with Pulmonary Alveolar Proteinosis. Medicine.

[44] Oerlemans W.G.H., Jansen E.N.H., Prevo R.L., Eijsvogel M.M.M. (2009). Primary cerebellar nocardiosis and alveolar proteinosis. Acta Neurol. Scand..

[45] Ranchod M., Bissell M. (1979). Pulmonary alveolar proteinosis and cytomegalovirus infection. Arch. Pathol. Lab. Med..

[46] Van Nhieu J.T., Vojtek A.-M., Bernaudin J.-F., Escudier E., Fleury-Feith J. (1990). Pulmonary Alveolar Proteinosis Associated with Pneumocystis carinii: Ultrastructural identification in bronchoalveolar lavage in AIDS and immunocompromised non-AIDS patients. Chest.

[47] Uchiyama M., Nagao T., Hattori A., Fujii T., Ichiwata T., Nakata K., Tani K., Hayashi T. (2009). Pulmonary alveolar proteinosis in a patient with Behcet’s disease. Respirology.

[48] Yamasaki M., Kawamoto K., Nakano S., Taniwaki M., Matsumoto N., Hattori N. (2021). Secondary pulmonary alveolar proteinosis in a patient with systemic lupus erythematosus. Pulmonology.

[49] Ceruti M., Rodi G., Stella G.M., Adami A., Bolongaro A., Baritussio A., Pozzi E., Luisetti M. (2007). Successful whole lung lavage in pulmonary alveolar proteinosis secondary to lysinuric protein intolerance: A case report. Orphanet J. Rare Dis..

[50] Cho K., Yamada M., Agematsu K., Kanegane H., Miyake N., Ueki M., Akimoto T., Kobayashi N., Ikemoto S., Tanino M. (2018). Heterozygous Mutations in OAS1 Cause Infantile-Onset Pulmonary Alveolar Proteinosis with Hypogammaglobulinemia. Am. J. Hum. Genet..

[51] Hadchouel A., Wieland T., Griese M., Baruffini E., Lorenz-Depiereux B., Enaud L., Graf E., Dubus J.C., Halioui-Louhaichi S., Coulomb A. (2015). Biallelic Mutations of Methionyl-tRNA Synthetase Cause a Specific Type of Pulmonary Alveolar Proteinosis Prevalent on Réunion Island. Am. J. Hum. Genet..

[52] Costabel U., Nakata K. (2010). Pulmonary Alveolar Proteinosis Associated with Dust Inhalation. Am. J. Respir. Crit. Care Med..

[53] Cummings K.J., Donat W.E., Ettensohn D.B., Roggli V.L., Ingram P., Kreiss K. (2010). Pulmonary Alveolar Proteinosis in Workers at an Indium Processing Facility. Am. J. Respir. Crit. Care Med..

[54] Takaki M., Tanaka T., Komohara Y., Tsuchihashi Y., Mori D., Hayashi K., Fukuoka J., Yamasaki N., Nagayasu T., Ariyoshi K. (2016). Recurrence of pulmonary alveolar proteinosis after bilateral lung transplantation in a patient with a nonsense mutation in CSF2RB. Respir. Med. Case Rep..

[55] Divithotawela C., Apte S.H., Tan M.E., De Silva T.A., Chambers D.C. (2020). Pulmonary alveolar proteinosis after lung transplantation. Respirol. Case Rep..

[56] Tokman S., Hahn M.F., Abdelrazek H., Panchabhai T.S., Patel V.J., Walia R., Omar A. (2016). Lung Transplant Recipient with Pulmonary Alveolar Proteinosis. Case Rep. Transplant..

[57] Kleff V., Sorg U.R., Bury C., Suzuki T., Rattmann I., Jerabek-Willemsen M., Poremba C., Flasshove M., Opalka B., Trapnell B. (2008). Gene therapy of beta(c)-deficient pulmonary alveolar proteinosis (beta(c)-PAP): Studies in a murine in vivo model. Mol. Ther..

[58] Hetzel M., Lopez-Rodriguez E., Mucci A., Nguyen A.H.H., Suzuki T., Shima K., Buchegger T., Dettmer S., Rodt T., Bankstahl J.P. (2019). Effective hematopoietic stem cell-based gene therapy in a murine model of hereditary pulmonary alveolar proteinosis. Haematologica.

[59] Nishinakamura R., Wiler R., Dirksen U., Morikawa Y., Arai K.I., Miyajima A., Burdach S., Murray R. (1996). The pulmonary alveolar proteinosis in granulocyte macrophage colony-stimulating factor/interleukins 3/5 beta c receptor-deficient mice is reversed by bone marrow transplantation. J. Exp. Med..

[60] Martinez-Moczygemba M., Doan M.L., Elidemir O., Fan L.L., Cheung S.W., Lei J.T., Moore J.P., Tavana G., Lewis L.R., Zhu Y. (2008). Pulmonary alveolar proteinosis caused by deletion of the GM-CSFRα gene in the X chromosome pseudoautosomal region. J. Exp. Med..

[61] Tanaka-Kubota M., Shinozaki K., Miyamoto S., Yanagimachi M., Okano T., Mitsuiki N., Ueki M., Yamada M., Imai K., Takagi M. (2017). Hematopoietic stem cell transplantation for pulmonary alveolar proteinosis associated with primary immunodeficiency disease. Int. J. Hematol..

[62] Van Lier Y.F., De Bree G.J., Jonkers R.E., Roelofs J.J., Berge I.J.T., Rutten C.E., Nur E., Kuijpers T.W., Hazenberg M.D., Zeerleder S.S. (2020). Allogeneic hematopoietic cell transplantation in the management of GATA2 deficiency and pulmonary alveolar proteinosis. Clin. Immunol..

[63] Ozcelik U., Aytac S., Kuskonmaz B., Yalcin E., Dogru D., Okur V., Kara A., Hizal M., Polat S.E., Emiralioglu N. (2021). Nonmyeloablative hematopoietic stem cell transplantation in a patient with hereditary pulmonary alveolar proteinosis. Pediatr. Pulmonol..

[64] Suzuki T., I Arumugam P., Sakagami T., Lachmann N., Chalk C., Sallese A., Abe S., Trapnell C., Carey B., Moritz T. (2014). Pulmonary macrophage transplantation therapy. Nat. Cell Biol..

[65] Mucci A., Lopez-Rodriguez E., Hetzel M., Liu S., Suzuki T., Happle C., Ackermann M., Kempf H., Hillje R., Kunkiel J. (2018). iPSC-derived macrophages effectively treat pulmonary alveolar proteinosis in Csf2rb-deficient mice. Stem Cell Rep..

[66] Happle C., Lachmann N., Kuljec J., Wetzke M., Ackermann M., Brennig S., Mucci A., Jirmo A.C., Groos S., Mirenska A. (2014). Pulmonary transplantation of macrophage progenitors as effective and long-lasting therapy for hereditary pulmonary alveolar proteinosis. Sci. Transl. Med..

[67] Happle C., Lachmann N., Ackermann M., Mirenska A., Göhring G., Thomay K., Mucci A., Hetzel M., Glomb T., Suzuki T. (2018). Pulmonary Transplantation of Human Induced Pluripotent Stem Cell–derived Macrophages Ameliorates Pulmonary Alveolar Proteinosis. Am. J. Respir. Crit. Care Med..

[68] Van De Laar L., Saelens W., De Prijck S., Martens L., Scott C.L., Van Isterdael G., Hoffmann E., Beyaert R., Saeys Y., Lambrecht B.N. (2016). Yolk Sac Macrophages, Fetal Liver, and Adult Monocytes Can Colonize an Empty Niche and Develop into Functional Tissue-Resident Macrophages. Immunity.

[69] Li F., Okreglicka K.M., Pohlmeier L.M., Schneider C., Kopf M. (2020). Fetal monocytes possess increased metabolic capacity and replace primitive macrophages in tissue macrophage development. EMBO J..

[70] Reed J.A., Ikegami M., Cianciolo E.R., Lu W., Cho P.S., Hull W., Jobe A.H., Whitsett J.A. (1999). Aerosolized GM-CSF ameliorates pulmonary alveolar proteinosis in GM-CSF-deficient mice. Am. J. Physiol. Cell. Mol. Physiol..

[71] Sheng G., Chen P., Wei Y., Chu J., Cao X., Zhang H.-L. (2018). Better approach for autoimmune pulmonary alveolar proteinosis treatment: Inhaled or subcutaneous granulocyte-macrophage colony-stimulating factor: A meta-analyses. Respir. Res..

[72] US National Library of Medicine (2020). ClinicalTrials.Gov. First in Human Study of Pioglitazone Therapy of Autoimmune Pulmonary Alveolar Proteinosis.

[73] McCarthy C., Lee E., Bridges J.P., Sallese A., Suzuki T., Woods J.C., Bartholmai B.J., Wang T., Chalk C., Carey B.C. (2018). Statin as a novel pharmacotherapy of pulmonary alveolar proteinosis. Nat. Commun..

[74] Ackermann M., Kempf H., Hetzel M., Hesse C., Hashtchin A.R., Brinkert K., Schott J.W., Haake K., Kühnel M.P., Glage S. (2018). Bioreactor-based mass production of human iPSC-derived macrophages enables immunotherapies against bacterial airway infections. Nat. Commun..

[75] Chen J., Su F.-Y., Das D., Srinivasan S., Son H.-N., Lee B., Radella F., Whittington D., Monroe-Jones T., West T.E. (2019). Glycan targeted polymeric antibiotic prodrugs for alveolar macrophage infections. Biomaterial.

[76] Su F.-Y., Srinivasan S., Lee B., Chen J., Convertine A.J., West T.E., Ratner D.M., Skerrett S.J., Stayton P.S. (2018). Macrophage-targeted drugamers with enzyme-cleavable linkers deliver high intracellular drug dosing and sustained drug pharmacokinetics against alveolar pulmonary infections. J. Control. Release.

[77] Chavas T.E., Su F.-Y., Srinivasan S., Roy D., Lee B., Lovelace-Macon L., Rerolle G.F., Limqueco E., Skerrett S.J., Ratner D.M. (2021). A macrophage-targeted platform for extending drug dosing with polymer prodrugs for pulmonary infection prophylaxis. J. Control. Release.

[78] Brinkert K., Hedtfeld S., Burhop A., Gastmeier R., Gad P., Wedekind D., Kloth C., Rothschuh J., Lachmann N., Hetzel M. (2021). Rescue from Pseudomonas aeruginosa Airway Infection via Stem Cell Transplantation. Mol. Ther..

[79] Hetzel M., Mucci A., Blank P., Nguyen A.H.H., Schiller J., Halle O., Kühnel M.-P., Billig S., Meineke R., Brand D. (2018). Hematopoietic stem cell gene therapy for IFNγR1 deficiency protects mice from mycobacterial infections. Blood.

[80] Humbert M., Taillé C., Mala L., Le Gros V., Just J., Molimard M. (2018). Omalizumab effectiveness in patients with severe allergic asthma according to blood eosinophil count: The STELLAIR study. Eur. Respir. J..

[81] Mellado M., de Ana A.M., Gómez L., Martínez-A C., Rodríguez-Frade J.M. (2007). Chemokine Receptor 2 Blockade Prevents Asthma in a Cynomolgus Monkey Model. J. Pharmacol. Exp. Ther..

[82] Careau E., Bissonnette E.Y. (2004). Adoptive Transfer of Alveolar Macrophages Abrogates Bronchial Hyperresponsiveness. Am. J. Respir. Cell Mol. Biol..

[83] Zhang F., Ayaub E.A., Wang B., Puchulu-Campanella E., Li Y., Hettiarachchi S.U., Lindeman S.D., Luo Q., Rout S., Srinivasarao M. (2020). Reprogramming of profibrotic macrophages for treatment of bleomycin-induced pulmonary fibrosis. EMBO Mol. Med..

[84] Gheberemedhin A., Salam A.B., Adu-Addai B., Noonan S., Stratton R., Ahmed M.S., Martin G., Huixian L., Andrews C., Balasubramanyam K. (2020). A novel CD206 targeting peptide inhibits bleomycin induced pulmonary fibrosis in mice. bioRxiv.

[85] Pan T., Zhou Q., Miao K., Zhang L., Wu G., Yu J., Xu Y., Xiong W., Li Y., Wang Y. (2021). Suppressing Sart1 to modulate macrophage polarization by siRNA-loaded liposomes: A promising therapeutic strategy for pulmonary fibrosis. Theranostics.

[86] Matsuhira T., Nishiyama O., Tabata Y., Kaji C., Kubota-Ishida N., Chiba Y., Sano H., Iwanaga T., Tohda Y. (2020). A novel phosphodiesterase 4 inhibitor, AA6216, reduces macrophage activity and fibrosis in the lung. Eur. J. Pharmacol..

[87] Dickson R.P., Erb-Downward J.R., Martinez F.J., Huffnagle G.B. (2016). The microbiome and the respiratory Tract. Annu. Rev. Physiol..

[88] Dickson R.P., Erb-Downward J.R., Freeman C.M., McCloskey L., Falkowski N.R., Huffnagle G.B., Curtis J.L. (2017). Bacterial Topography of the Healthy Human Lower Respiratory Tract. mBio.

[89] Puttur F., Gregory L.G., Lloyd C.M. (2019). Airway macrophages as the guardians of tissue repair in the lung. Immunol. Cell Biol..

[90] Janssen W.J., Stefanski A.L., Bochner B.S., Evans C.M. (2016). Control of lung defence by mucins and macrophages: Ancient defence mechanisms with modern functions. Eur. Respir. J..

[91] Ali F., Pozzi G., Lee M.E., Iannelli F., Mitchell T., Read R.C., Dockrell D.H. (2003). Streptococcus pneumoniae–Associated Human Macrophage Apoptosis after Bacterial Internalization via Complement and Fcγ Receptors Correlates with Intracellular Bacterial Load. J. Infect. Dis..

[92] Aberdein J.D., Cole J., Bewley M., Dockrell D.H., Marriott H.M. (2013). Alveolar macrophages in pulmonary host defence- the unrecognised role of apoptosis as a mechanism of intracellular bacterial killing. Clin. Exp. Immunol..

[93] Büll C., Heise T., Adema G.J., Boltje T.J. (2016). Sialic Acid Mimetics to Target the Sialic Acid–Siglec Axis. Trends Biochem. Sci..

[94] Chang Y.-C., Olson J., Beasley F.C., Tung C., Zhang J., Crocker P.R., Varki A., Nizet V. (2014). Group B Streptococcus Engages an Inhibitory Siglec through Sialic Acid Mimicry to Blunt Innate Immune and Inflammatory Responses In Vivo. PLOS Pathog..

[95] Foster T.J. (2005). Immune evasion by staphylococci. Nat. Rev. Genet..

[96] Guidi-Rontani C. (2002). The alveolar macrophage: The Trojan horse of Bacillus anthracis. Trends Microbiol..

[97] Lacoma A., Cano V., Moranta D., Regueiro V., Domínguez-Villanueva D., Laabei M., González-Nicolau M., Ausina V., Prat C., Bengoechea J.A. (2017). Investigating intracellular persistence ofStaphylococcus aureuswithin a murine alveolar macrophage cell line. Virulence.

[98] Rajaram M.V.S., Brooks M.N., Morris J.D., Torrelles J.B., Azad A.K., Schlesinger L.S. (2010). Mycobacterium tuberculosisActivates Human Macrophage Peroxisome Proliferator-Activated Receptor γ Linking Mannose Receptor Recognition to Regulation of Immune Responses. J. Immunol..

[99] Singh K.H., Jha B., Dwivedy A., Choudhary E.N.A.G., Ashraf A., Arora D., Agarwal N., Biswal B.K. (2017). Characterization of a secretory hydrolase from Mycobacterium tuberculosis sheds critical insight into host lipid utilization by M. tuberculosis. J. Biol. Chem..

[100] Silva-Gomes S., Vale-Costa S., Appelberg R., Gomes M.S. (2013). Iron in intracellular infection: To provide or to deprive?. Front. Cell. Infect. Microbiol..

[101] Vignesh K.S., Figueroa J.A.L., Porollo A., Divanovic S., Caruso J.A., Deepe G.S. (2016). IL-4 Induces Metallothionein 3- and SLC30A4-Dependent Increase in Intracellular Zn 2+ that Promotes Pathogen Persistence in Macrophages. Cell Rep..

[102] Nakamura S., Davis K.M., Weiser J.N. (2011). Synergistic stimulation of type I interferons during influenza virus coinfection promotes Streptococcus pneumoniae colonization in mice. J. Clin. Investig..

[103] Robinson K.M., McHugh K.J., Mandalapu S., Clay M.E., Lee B., Scheller E.V., Enelow R.I., Chan Y.R., Kolls J.K., Alcorn J.F. (2013). Influenza A virus exacerbates Staphylococcus aureus pneumonia in mice by attenuating antimicrobial peptide production. J. Infect. Dis..

[104] Mehta D., Petes C., Gee K., Basta S. (2015). The Role of Virus Infection in Deregulating the Cytokine Response to Secondary Bacterial Infection. J. Interf. Cytokine Res..

[105] Sun K., Metzger D.W. (2008). Inhibition of pulmonary antibacterial defense by interferon-γ during recovery from influenza infection. Nat. Med..

[106] Ghoneim H.E., Thomas P.G., McCullers J.A. (2013). Depletion of Alveolar Macrophages during Influenza Infection Facilitates Bacterial Superinfections. J. Immunol..

[107] Tobin J.M., Nickolich K.L., Ramanan K., Pilewski M.J., Lamens K.D., Alcorn J.F., Robinson K.M. (2020). Influenza Suppresses Neutrophil Recruitment to the Lung and Exacerbates Secondary Invasive Pulmonary Aspergillosis. J. Immunol..

[108] Roquilly A., Jacqueline C., Davieau M., Mollé A., Sadek A., Fourgeux C., Rooze P., Broquet A., Misme-Aucouturier B., Chaumette T. (2020). Alveolar macrophages are epigenetically altered after inflammation, leading to long-term lung immunoparalysis. Nat. Immunol..

[109] Morgan D.J., Casulli J., Chew C., Connolly E., Lui S., Brand O.J., Rahman R., Jagger C., Hussell T. (2018). Innate Immune Cell Suppression and the Link With Secondary Lung Bacterial Pneumonia. Front. Immunol..

[110] Kachroo A., Kachroo P. (2020). Mobile signals in systemic acquired resistance. Curr. Opin. Plant Biol..

[111] Kurtz J. (2005). Specific memory within innate immune systems. Trends Immunol..

[112] van der Meer J.W., Joosten L.A., Riksen N., Netea M.G. (2015). Trained immunity: A smart way to enhance innate immune defence. Mol. Immunol..

[113] Bustamante J. (2020). Mendelian susceptibility to mycobacterial disease: Recent discoveries. Qual. Life Res..

[114] Elborn J.S. (2016). Cystic fibrosis. Lancet.

[115] Di A., Brown M.E., Deriy L.V., Li C., Szeto F.L., Chen Y., Huang P., Tong J., Naren A.P., Bindokas V. (2006). CFTR regulates phagosome acidification in macrophages and alters bactericidal activity. Nat. Cell Biol..

[116] Zhang S.-Y., Zhang Q., Casanova J.-L., Su H.C., Covid the COVID Team (2020). Severe COVID-19 in the young and healthy: Monogenic inborn errors of immunity?. Nat. Rev. Immunol..

[117] Zhang Q., Bastard P., Liu Z., Le Pen J., Moncada-Velez M., Chen J., Ogishi M., Sabli I.K.D., Hodeib S., Korol C. (2020). Inborn errors of type I IFN immunity in patients with life-threatening COVID. Science.

[118] Pairo-Castineira E., Clohisey S., Klaric L., Bretherick A.D., Rawlik K., Pasko D., Walker S., Parkinson N., Fourman M.H., Russell C.D. (2021). Genetic mechanisms of critical illness in COVID. Nat. Cell Biol..

[119] Bastard P., Rosen L.B., Zhang Q., Michailidis E., Hoffmann H.-H., Zhang Y., Dorgham K., Philippot Q., Rosain J., Béziat V. (2020). Auto-antibodies against type I IFNs in patients with life-threatening COVID. Science.

[120] Dalskov L., Møhlenberg M., Thyrsted J., Blay-Cadanet J., Poulsen E.T., Folkersen B.H., Skaarup S.H., Olagnier D., Reinert L., Enghild J.J. (2020). SARS-CoV-2 evades immune detection in alveolar macrophages. EMBO Rep..

[121] Tapela K., Olwal C.O., Quaye O. (2020). Parallels in the pathogenesis of SARS-CoV-2 and M. tuberculosis: A synergistic or antagonistic alliance?. Future Microbiol..

[122] Song X., Hu W., Yu H., Zhao L., Zhao Y., Zhao Y. (2020). High expression of angiotensin-converting enzyme-2 (ACE2) on tissue macrophages that may be targeted by virus SARS-CoV-2 in COVID-19 patients. bioRxiv.

[123] Ortiz M.E., Thurman A., Pezzulo A.A., Leidinger M.R., Klesney-Tait J.A., Karp P.H., Tan P., Wohlford-Lenane C., McCray P.B., Meyerholz D.K. (2020). Heterogeneous expression of the SARS-Coronavirus-2 receptor ACE2 in the human respiratory tract. EBioMedicine.

[124] Saffern M. (2020). ACE2 is not induced by interferon. Nat. Rev. Immunol..

[125] Abassi Z., Knaney Y., Karram T., Heyman S.N. (2020). The Lung Macrophage in SARS-CoV-2 Infection: A Friend or a Foe?. Front. Immunol..

[126] Agustí A., Hogg J.C. (2019). Update on the Pathogenesis of Chronic Obstructive Pulmonary Disease. N. Engl. J. Med..

[127] Lafuse W.P., Rajaram M.V.S., Wu Q., Moliva J.I., Torrelles J.B., Turner J., Schlesinger L.S. (2019). Identification of an Increased Alveolar Macrophage Subpopulation in Old Mice That Displays Unique Inflammatory Characteristics and Is Permissive toMycobacterium tuberculosisInfection. J. Immunol..

[128] Veldhuizen R.A.W., McCaig L.A., Pape C., Gill S.E. (2019). The effects of aging and exercise on lung mechanics, surfactant and alveolar macrophages. Exp. Lung Res..

[129] Falgenhauer L., Waezsada S.-E., Yao Y., Imirzalioglu C., Käsbohrer A., Roesler U., Michael G.B., Schwarz S., Werner G., Kreienbrock L. (2016). Colistin resistance gene mcr-1 in extended-spectrum β-lactamase-producing and carbapenemase-producing Gram-negative bacteria in Germany. Lancet Infect. Dis..

[130] Gould I. (2013). Treatment of bacteraemia: Meticillin-resistant Staphylococcus aureus (MRSA) to vancomycin-resistant S. aureus (VRSA). Int. J. Antimicrob. Agents.

[131] Mehta M., Deeksha A., Sharma N., Vyas M., Khurana N., Maurya P.K., Singh H., de Jesus T.P.A., Dureja H., Chellappan D.K. (2019). Interactions with the macrophages: An emerging targeted approach using novel drug delivery systems in respiratory diseases. Chem. Interact..

[132] Pham D.-D., Fattal E., Tsapis N. (2015). Pulmonary drug delivery systems for tuberculosis treatment. Int. J. Pharm..

[133] Shivangi A., Meena L.S. (2018). A Novel Approach in Treatment of Tuberculosis by Targeting Drugs to Infected Macrophages Using Biodegradable Nanoparticles. Appl. Biochem. Biotechnol..

[134] Draijer C., Peters-Golden M. (2017). Alveolar Macrophages in Allergic Asthma: The Forgotten Cell Awakes. Curr. Allergy Asthma Rep..

[135] Zasłona Z., Przybranowski S., Wilke C., Van Rooijen N., Teitz-Tennenbaum S., Osterholzer J.J., Wilkinson J.E., Moore B.B., Peters-Golden M. (2014). Resident Alveolar Macrophages Suppress, whereas Recruited Monocytes Promote, Allergic Lung Inflammation in Murine Models of Asthma. J. Immunol..

[136] Lee Y.G., Jeong J.J., Nyenhuis S., Berdyshev E., Chung S., Ranjan R., Karpurapu M., Deng J., Qian F., Kelly E.A.B. (2015). Recruited Alveolar Macrophages, in Response to Airway Epithelial–Derived Monocyte Chemoattractant Protein 1/CCL2, Regulate Airway Inflammation and Remodeling in Allergic Asthma. Am. J. Respir. Cell Mol. Biol..

[137] Machiels B., Dourcy M., Xiao X., Javaux J., Mesnil C., Sabatel C., Desmecht D., Lallemand F., Martinive P., Hammad H. (2017). A gammaherpesvirus provides protection against allergic asthma by inducing the replacement of resident alveolar macrophages with regulatory monocytes. Nat. Immunol..

[138] Fricker M., Gibson P.G. (2017). Macrophage dysfunction in the pathogenesis and treatment of asthma. Eur. Respir. J..

[139] Liang Z., Zhang Q., Thomas C.M., Chana K.K., Gibeon D., Barnes P.J., Chung K.F., Bhavsar P.K., Donnelly L.E. (2014). Impaired macrophage phagocytosis of bacteria in severe asthma. Respir. Res..

[140] Fitzpatrick A.M., Holguin F., Teague W.G., Brown L.A.S. (2008). Alveolar macrophage phagocytosis is impaired in children with poorly controlled asthma. J. Allergy Clin. Immunol..

[141] Fitzpatrick A.M., Teague W.G., Burwell L., Brown M.S., Brown L.A.S. (2011). NIH/NHLBI Severe Asthma Research Program Glutathione Oxidation Is Associated With Airway Macrophage Functional Impairment in Children With Severe Asthma. Pediatr. Res..

[142] Aran D., Looney A.P., Liu L., Wu E., Fong V., Hsu A., Chak S., Naikawadi R.P., Wolters P.J., Abate A.R. (2019). Reference-based analysis of lung single-cell sequencing reveals a transitional profibrotic macrophage. Nat. Immunol..

[143] Reader B.F., Sethuraman S., Hay B.R., Becket R.V.T., Karpurapu M., Chung S., Lee Y.G., Christman J.W., Ballinger M.N. (2020). IRAK-M Regulates Monocyte Trafficking to the Lungs in Response to Bleomycin Challenge. J. Immunol..

[144] Nakazawa Y., Ohtsuka S., Nakahashi-Oda C., Shibuya A. (2019). Cutting Edge: Involvement of the Immunoreceptor CD300c2 on Alveolar Macrophages in Bleomycin-Induced Lung Fibrosis. J. Immunol..

[145] Zhang N., Yang K., Bai J., Yi J., Gao C., Zhao J., Liang S., Wei T., Feng L., Song L. (2020). Myeloid-specific blockade of Notch signaling alleviates murine pulmonary fibrosis through regulating monocyte-derived Ly6c lo MHCII hi alveolar macrophages recruitment and TGF-β secretion. FASEB J..

[146] Misharin A.V., Morales-Nebreda L., Reyfman P.A., Cuda C.M., Walter J.M., McQuattie-Pimentel A.C., Chen C.-I., Anekalla K.R., Joshi N., Williams K.J. (2017). Monocyte-derived alveolar macrophages drive lung fibrosis and persist in the lung over the life span. J. Exp. Med..

[147] Guilliams M., Thierry G.R., Bonnardel J., Bajenoff M. (2020). Establishment and Maintenance of the Macrophage Niche. Immunity.

[148] Reyfman P.A., Walter J.M., Joshi N., Anekalla K.R., McQuattie-Pimentel A.C., Chiu S., Fernandez R., Akbarpour M., Chen C.-I., Ren Z. (2019). Single-Cell Transcriptomic Analysis of Human Lung Provides Insights into the Pathobiology of Pulmonary Fibrosis. Am. J. Respir. Crit. Care Med..

[149] Allard B., Panariti A., Martin J.G. (2018). Alveolar Macrophages in the Resolution of Inflammation, Tissue Repair, and Tolerance to Infection. Front. Immunol..

[150] Allden S.J., Ogger P.P., Ghai P., McErlean P., Hewitt R., Toshner R., Walker S.A., Saunders P., Kingston S., Molyneaux P.L. (2019). The Transferrin Receptor CD71 Delineates Functionally Distinct Airway Macrophage Subsets during Idiopathic Pulmonary Fibrosis. Am. J. Respir. Crit. Care Med..

[151] Vlahos R., Bozinovski S. (2014). Role of alveolar macrophages in chronic obstructive pulmonary disease. Front. Immunol..

[152] Cosío B.G., Mann B., Ito K., Jazrawi E., Barnes P.J., Chung K.F., Adcock I.M. (2004). Histone Acetylase and Deacetylase Activity in Alveolar Macrophages and Blood Mononocytes in Asthma. Am. J. Respir. Crit. Care Med..

[153] Busse W., Corren J., Lanier B.Q., McAlary M., Fowler-Taylor A., Della C.G., Van As A., Gupta N. (2001). Omalizumab, anti-IgE recombinant humanized monoclonal antibody, for the treatment of severe allergic asthma. J. Allergy Clin. Immunol..

[154] Suraya R., Nagano T., Katsurada M., Sekiya R., Kobayashi K., Nishimura Y. (2021). Molecular mechanism of asthma and its novel molecular target therapeutic agent. Respir. Investig..

[155] Raghu G., Selman M. (2015). Nintedanib and Pirfenidone. New Antifibrotic Treatments Indicated for Idiopathic Pulmonary Fibrosis Offer Hopes and Raises Questions. Am. J. Respir. Crit. Care Med..

[156] Buchrieser J., James W., Moore M.D. (2017). Human Induced Pluripotent Stem Cell-Derived Macrophages Share Ontogeny with MYB -Independent Tissue-Resident Macrophages. Stem Cell Rep..

[157] Litvack M.L., Wigle T.J., Lee J., Wang J., Ackerley C., Grunebaum E., Post M. (2016). Alveolar-like Stem Cell–derivedMyb−Macrophages Promote Recovery and Survival in Airway Disease. Am. J. Respir. Crit. Care Med..

